# A new integrative approach combining right heart catheterization and echocardiography to stage aortic stenosis-related cardiac damage

**DOI:** 10.3389/fcvm.2023.1184308

**Published:** 2023-08-02

**Authors:** Tommaso Viva, Adriana Postolache, Mai-Linh Nguyen Trung, Pauline Danthine, Hélène Petitjean, Vito Domenico Bruno, Christophe Martinez, Mathieu Lempereur, Marco Guazzi, Samy Aghezzaf, Augustin Coisne, Cécile Oury, Raluca Dulgheru, Patrizio Lancellotti

**Affiliations:** ^1^ GIGA Cardiovascular Sciences, CHU Sart Tilman, Cardiology Department, University of Liège Hospital, Liège, Belgium; ^2^ Department of Biomedical Sciences for Health, University of Milano, Milan, Italy; ^3^ Department of Minimally Invasive Cardiac Surgery, IRCCS Galeazzi—Sant’Ambrogio Hospital, Milan, Italy; ^4^ School of Medicine, Department of Biological Sciences, University of Milano, Milan, Italy; ^5^ Cardiology Division, San Paolo Hospital, Milan, Italy; ^6^ CHU Lille, Institut Pasteur de Lille, University Lille, Inserm, Lille, France; ^7^ Cardiovascular Research Foundation, New York, NY, United States; ^8^ Gruppo Villa Maria Care and Research, Maria Cecilia Hospital, Cotignola, and Anthea Hospital, Bari, Italy

**Keywords:** staging, right heart catheterization, echocardiography, aortic stenosis, TAVR

## Abstract

**Introduction:**

Although staging of the extent of aortic stenosis (AS)-related cardiac damages is usually performed via echocardiography, this technique has considerable limitations in assessing pulmonary artery and right chamber pressures. The present hypothesis-generating study sought to explore the efficacy of a staging system of cardiac damage based on echocardiographic and invasive [right heart catheterization (RHC)] hemodynamic parameters in patients undergoing transcatheter aortic valve implantation (TAVI).

**Methods:**

We studied 90 symptomatic patients with severe AS in whom echocardiographic and invasive evaluation by RHC was obtained prior to TAVI. Cardiac damage stages were defined as follows: no cardiac damage (stage 0), left ventricular (LV) damage (stage 1), left atrial or mitral valve damage (stage 2), pulmonary vasculature or tricuspid valve damage (stage 3), and right ventricular (RV) dysfunction or low-flow state (stage 4). With the integrative approach using RHC, pulmonary hypertension (PH) was defined as an mPAP ≥25 mmHg and the low-flow state corresponded to a cardiac index of <1.8 L/min/m^2^ and a right atrial pressure of >10 mmHg.

**Results:**

During follow-up (median: 2.9 years), 43 patients (47.8%) died. The integrative cardiac damage staging was associated with a significant increase in all-cause and cardiovascular mortality per each increase of cardiac damage stage, whereas the outcome was similar according to the echocardiographic staging.

**Conclusions:**

A staging system of cardiac lesion based on echocardiographic and invasive hemodynamic parameters in patients with severe AS undergoing TAVI predicts mortality. Patients with pre-existing PH, ≥ moderate tricuspid regurgitation and/or RV dysfunction, and a low-flow state had a markedly increased risk of death. Further larger studies are needed to validate our findings.

## 1. Introduction

In the western world, calcific aortic stenosis (AS), the most common valvular heart disease, represents a major public health burden (1). Current indications for aortic valve replacement (AVR) are based on the severity of AS (aortic pressure gradients, aortic valve area) and the presence of symptoms or of left ventricular (LV) dysfunction (LV ejection fraction <50%) (2). However, the clinical consequences of AS result not only from the valvular obstruction itself, but also from the progressive changes in LV structure (hypertrophy, remodeling) and function (intrinsic myocardial dysfunction) with subsequent diastolic dysfunction, elevated left atrial pressures, left atrium dilation, pulmonary hypertension (PH), tricuspid regurgitation, and right ventricular (RV) dysfunction (3). All these structural and functional changes reflect the extent of AS-related cardiac damages, which has a significant impact on patient prognosis. Staging of the extent of AS-related cardiac damages is usually performed by echocardiography (4–9), but this technique has considerable limitations in assessing pulmonary and right heart pressures. Although the presence of PH is a major prognostic determinant, current guidelines do not recommend the routine performance of right heart catheterization (RHC) in the workup of patients with AS. Maeder et al. (10) have recently pointed out that using a staging system of cardiac damage based solely on invasive hemodynamic parameters in patients with severe AS undergoing AVR could also serve as a model for predicting post-procedural mortality. In daily practice, the role of the RHC in conjunction with non-invasive testing is increasingly used in the assessment of patients with PH in contrast to what occurs in AS patients. It is unknown whether the use of an integrative cardiac damage staging system based on the combined findings of echocardiography and invasive RHC may have clinical value. The present study sought to assess the usefulness of a combined invasive and non-invasive staging system to define the extent of AS-related cardiac injury in patients undergoing transcatheter aortic valve implantation (TAVI).

## 2. Materials and methods

### 2.1. Study population

**Figure 1:**
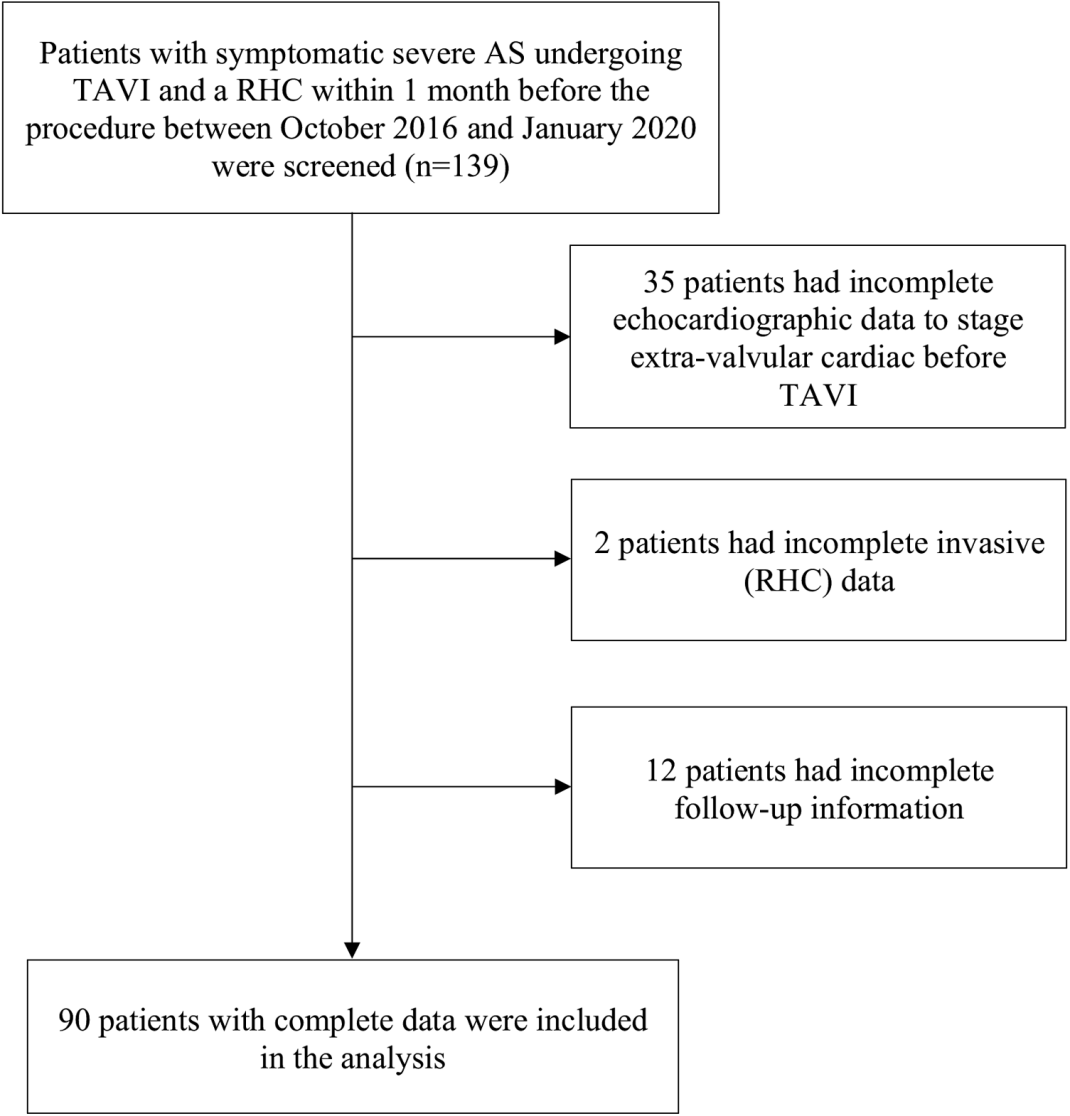
Study flowchart. AS, aortic stenosis; TAVI, transcatheter aortic valve implantation; RHC, right heart catheterization.

We conducted a retrospective analysis of systematically collected data of symptomatic patients with severe AS who underwent TAVI and who had RHC within 1 month prior to intervention in our Heart Valve Center (Department of Cardiology and Cardiovascular Surgery, CHU de Liege) between October 2016 and January 2020. A total of 139 symptomatic patients were screened during the study period. After the exclusion of patients who underwent valve-in-valve TAVI procedure, patients with moderate to severe mitral stenosis, patients with incomplete echocardiographic or RHC data, and patients who lacked follow-up information, the final analysis consisted of 90 patients (Figure 1). Baseline demographic and clinical data were collected using the regional health department information system and analyzed. The study was conducted in accordance with the Declaration of Helsinki and approved by the Ethics Committee of Liege University Hospital (protocol code: 2021/306, date of approval: 12 October 2021).

### 2.2. Right heart catheterization

RHC was performed with a 7Fr Swan–Ganz catheter by femoral access. Right atrial pressure, systolic (sPAP), diastolic, and mean (mPAP) pulmonary arterial pressure and pulmonary capillary wedge pressure were measured. Cardiac output was estimated through the thermodilution method. From these data, we derived pulmonary vascular resistance [(mPAP—pulmonary capillary wedge pressure)/cardiac output], cardiac index (cardiac output/body surface area), and stroke volume index (cardiac index/heart rate). PH was defined and classified according to the 2015 European guidelines, which was the existing recommendations on PH at the time the study was conducted (11).

### 2.3. Echocardiography

Transthoracic echocardiography with a Vivid 95 GE machine was performed by a cardiologist with high experience in valvular heart disease assessment. Echocardiographic images were analyzed using the EchoPac software v204 (GE Vingmed Ultrasound AS, Horten, Norway). The presence of severe AS, the chambers dimensions, LV and RV function, and valvular regurgitation evaluation were defined according to the current guidelines (2, 12). LV diastolic dysfunction was evaluated according to the latest guidelines (13). The sPAP was estimated adding the RV systolic pressure, calculated from the peak velocity of the tricuspid regurgitant jet according to the simplified Bernoulli equation, to the RAP, determined by the inspiratory collapse and the diameter of the inferior vena cava (14).

### 2.4. Stages of cardiac damage

**Figure 2:**
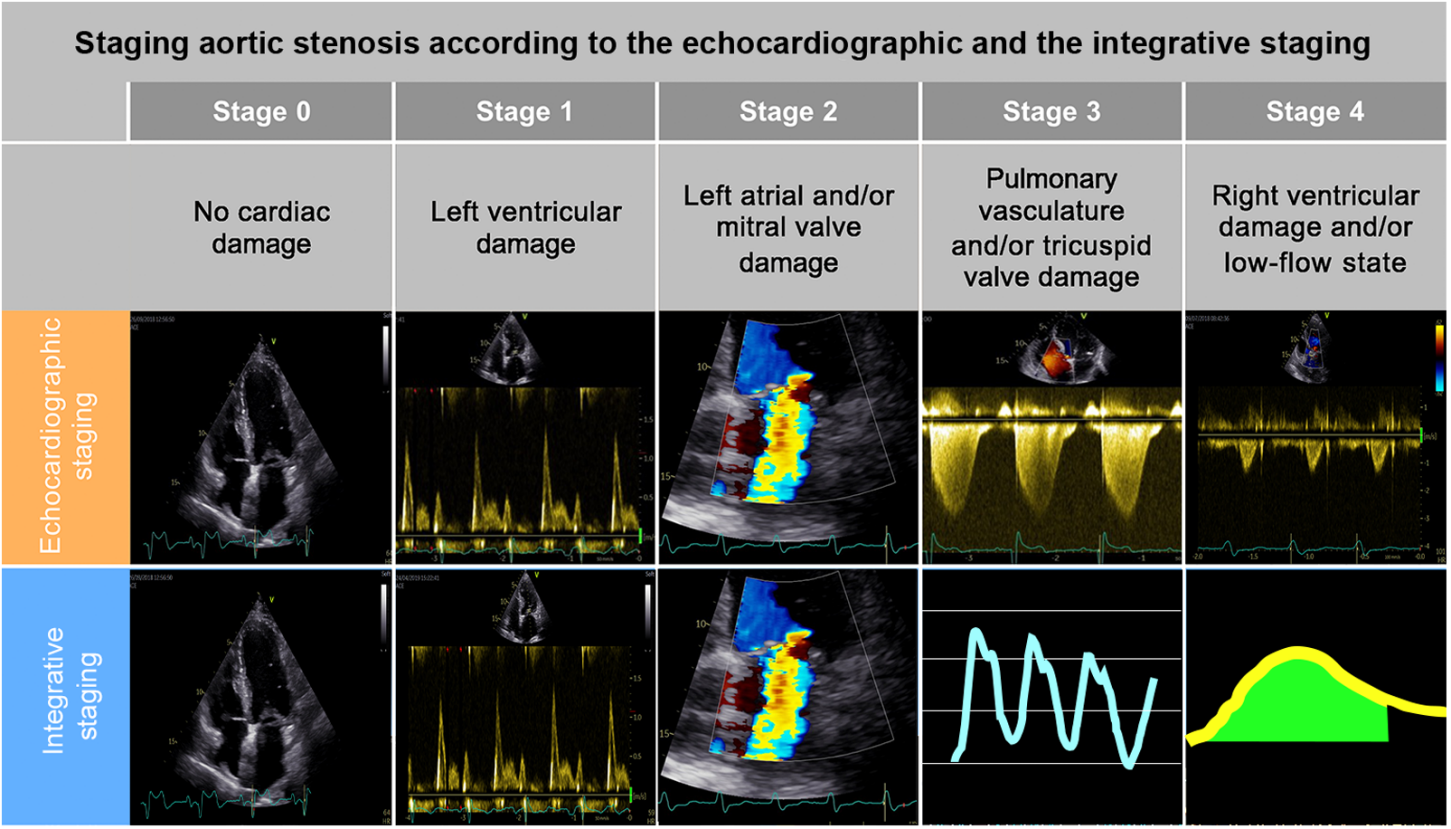
Classification according to the two analyzed aortic stenosis staging models and their application in survival probability. A comparison between the main parameters included in the current echocardiographic AS staging (top): stage 0: normal left and right chambers (no cardiac damage); stage 1: restrictive mitral inflow pattern (LV diastolic dysfunction grade 3) with a pathological L-wave during diastasis (LV damage); stage 2: moderate mitral regurgitation (mitral valve damage); stage 3: continuous wave Doppler signal used to calculate RV systolic pressure from the peak velocity of the tricuspid regurgitant jet according to the simplified Bernoulli equation (pulmonary vasculature damage); stage 4: pulsed wave Doppler in LV outflow tract to estimate LV stroke volume (low-flow state) and the proposed integrative staging model (bottom): stages 0,1, 2 as described above, stage 3: pulmonary artery wave pressure tracings during RHC (pulmonary vasculature damage), stage 4: thermodilution method to measure cardiac output during RHC (low-flow state).

The patients were categorized into five stages according to the extent of extra-aortic valve cardiac damage based solely on echocardiographic data (the echocardiographic staging) or based on combined echocardiographic and RHC data (the integrative staging) (Figure 2):

- The echocardiographic staging was based on the one proposed by Tastet et al. (6): stage 0: no cardiac damage; stage 1, LV damage: LV hypertrophy (LV mass index >95 g/m^2^ for women, >115 g/m^2^ for men), and/or LV diastolic dysfunction ≥ grade 2 and/or LV systolic dysfunction (LV ejection fraction <60%); stage 2, left atrial and/or mitral valve damage: left atrial enlargement (left atrium volume index >34 ml/m^2^) and/or ≥ moderate mitral regurgitation, and/or atrial fibrillation; stage 3, pulmonary vasculature or tricuspid valve damage: PH defined as sPAP ≥ 60 mmHg, and/or ≥ moderate tricuspid regurgitation; stage 4, RV damage and/or subclinical heart failure: RV dysfunction based on a multiparametric evaluation (TAPSE <17 mm and s′ < 9.5 cm/s and fractional area change <35%) and/or low-flow state (stroke volume index <30 ml/m^2^).
- In the integrative cardiac damage staging, the definitions of PH and of low-flow state were based on RHC data: stages 0,1, and 2 were assessed by echocardiography as aforementioned, whereas stages 3 and 4 were assessed integrating the data from RHC: stage 3: PH was defined by a mPAP ≥ 25 mmHg at RHC, and/or ≥ moderate tricuspid regurgitation on echocardiography; stage 4: RV dysfunction (TAPSE <17 mm and s′ <9.5 cm/s and fractional area change <35%) and/or low-flow state, defined at RHC by cardiac index <1.8 L/min/m^2^ and right atrial pressure >10 mmHg).

In both classifications, the patients were hierarchically classified in a given stage (worst stage) if at least one of the proposed criteria was satisfied. Given the small number of patients observed in stages 0–1, we described our population by merging stages 0–1–2, corresponding to left-chamber cardiac damage.

### 2.5. Clinical follow-up and end-point assessment

After TAVI, the patients were routinely followed-up and managed according to the available guidelines. Clinical endpoints were obtained from medical reports. Primary outcome was all-cause death. Secondary outcomes were the occurrence of cardiovascular death, stroke, myocardial infarction, and heart failure hospitalization.

### 2.6. Statistical analysis

Qualitative variables are presented as count and percentage and comparisons were performed by chi-square test or Fisher exact test when appropriate. Quantitative variables are presented as mean ± SD, and comparisons were performed by ANOVA test. To compare the survival function of different cardiac damage stage, the time-to-event data Kaplan–Meier plots and log-rank test were used. A pair-wise comparison between group levels for log-rank test was conducted with corrections for multiple testing using the Benjamini–Hochberg methods. To investigate the independent association between mortality and the new staging classification, univariable and multivariable Cox proportional hazards model were developed and adjusted for age, New York Heart Association (NYHA) class, chronic kidney disease, diabetes, history of atrial fibrillation, and peak aortic valve velocity according to significance in univariable analysis or as known risk factor. All statistical analyses were performed two-sided with SAS 9.4 (SAS Institute, Cary, NC, USA) and R (version 4.2.2—R core team. R: A language and environment for statistical computing. R Foundation for statistical computing, Vienna, Austria, URL https://www.R-project.org/), and a *p*-value < 0.05 was considered statistically significant.

## 3. Results

### 3.1. Baseline demographic, clinical and procedural characteristics

Tables 1, 2 depict the prevalence of the two cardiac damage staging models. The mean age of our population was 82.2 ± 5.5 years. According to the echocardiographic staging, the patients in stage 4 were slightly younger than the patients classified in the other stages (*p* = 0.03). Female represented 55.6% of the total population, and no significant gender differences were present between groups (Supplementary Material). Most of the patients had hypertension (91.1%) and dyslipidemia (74.4%), one-third had diabetes (32.2%), and more than one-half had coronary artery disease (65.6%). Cardiac risk factors and past medical history were comparable between groups. The mean STS score was 4.9 ± 2.7 with no significant difference between groups. The patients in advanced stages had more frequently atrial fibrillation. A large part of the population was in NYHA functional class III/IV (54.4%). In the echocardiographic staging, only the patients in stage 4 were more frequently in NYHA class III/IV (40.0% of the patients in stages 0–2 and in stage 3 were in NYHA class III/IV, whereas 74.4% of the patients in stage 4 were in NYHA class III/IV; *p* = 0.01), while in the integrative staging, stages 3 and 4 had a similar percentage of patients in NYHA class III/IV (43.2% in stages 0–2, 60.0% in stage 3, 68.2% in stage 4; *p* = 0.14).

**Table 1:** Prevalence of cardiac stages and their individual components according to the echocardiographic staging.

Stages of cardiac damage	
Stage 0 (no cardiac damage)	1/90 (1.1%)
Stage 1 (LV damage)	5/90 (5.5%)
Stage 2 (LA/MV damage)	35/90 (38.9%)
Stage 3 (pulmonary vasculature/TV damage)	10/90 (11.1%)
Stage 4 (RV damage/low-flow state)	39/90 (43.3%)
Individual components of cardiac damage types among the study population	
Stage 1: LV damage	81/90 (90.0%)
Increased LV mass index (>115 g/m^2^ male, >95 female g/m^2^)	61/90 (68.0%)
LV diastolic dysfunction grade ≥ 2	46/90 (51.1%)
Subclinical LV systolic dysfunction (LVEF < 60%)	53/90 (58.9%)
Stage 2: LA/MV damage	76/90 (84.4%)
Indexed left atrial volume >34 ml/m^2^	67/90 (74.4%)
Mitral regurgitation ≥ moderate	17/90 (18.9%)
Atrial fibrillation	40/90 (44.4%)
Stage 3: Pulmonary vasculature/TV damage	27 (30.0%)
Pulmonary hypertension (sPAP ≥ 60 mmHg)	20 (22.2%)
Tricuspid regurgitation ≥ moderate	18/90 (20.0%)
Stage 4: RV damage/low-flow state	26/90 (28.8%)
RV dysfunction	21/90 (38.8%)
Low-flow state (SVi < 30 ml/m^2^)	8/90 (8.9%)

LV, left ventricular; LA, left atrial; MV, mitral valve; TV, tricuspid valve; RV, right ventricular; sPAP, systolic pulmonary arterial pressure; SVi, stroke volume index.

**Table 2:** Prevalence of cardiac stages and their individual components according to the integrative staging.

Stages of cardiac damage	
Stage 0 (no cardiac damage)	2/90 (2.2%)
Stage 1 (LV damage)	7/90 (7.8%)
Stage 2 (LA/MV damage)	29/90 (32.2%)
Stage 3 (Pulmonary vasculature/TV damage)	30/90 (33.3%)
Stage 4 (RV damage/low-flow state)	22/90 (24.4%)
Individual components of cardiac damage types among the study population	
Stage 1: LV damage	81/90 (90.0%)
Increased LV mass index (>115 g/m^2^ male, >95 female g/m^2^)	61/90 (68.0%)
LV diastolic dysfunction grade ≥ 2	46/90 (51.1%)
Subclinical LV systolic dysfunction (LVEF < 60%)	53/90 (58.9%)
Stage 2: LA/MV damage	76/90 (84.4%)
Indexed LA volume >34 ml/m^2^	67/90 (74.4%)
Mitral regurgitation ≥ moderate	17/90 (18.9%)
Atrial fibrillation	40/90 (44.4%)
Stage 3: Pulmonary vasculature/TV damage	47/90 (52.2%)
Pulmonary hypertension (mPAP ≥ 25 mmHg)	41/90 (45.6%)
Moderate–severe tricuspid regurgitation	18/90 (20.0%)
Stage 4: RV damage/low-flow state	22/90 (24.4%)
RV dysfunction	21/90 (38.8%)
Low-flow state (CI < 1.8 L/min/m^2^ and RAP > 10 mmHg)	2/90 (2.2%)

LV, left ventricular; LA, left atrial; MV, mitral valve; TV, tricuspid valve; RV, right ventricular; mPAP, mean pulmonary arterial pressure; CI, cardiac index; RAP, right atrial pressure.

Intra-procedural and post-procedural characteristics of the patients are reported in Supplementary Table S2. No differences were found between the groups according to the two different staging models.

### 3.2. Baseline imaging data

Baseline pre-TAVI echocardiographic data for the entire population and according to the stage of cardiac involvement are summarized in Table 3. The mean LV mass index was 124.3 ± 36.6 g/m^2^, LV ejection fraction 54.7 ± 10.7%, stroke volume index 42.3 ± 9.6 ml/m^2^, peak aortic valve velocity 4.2 ± 0.7 m/s, aortic mean gradient 45.9 ± 15.0 mmHg, and aortic valve area 0.75 ± 0.16 cm^2^. In both staging classifications, the stage 4 patients had lower systolic blood pressure, possibly related to their low-flow state.

**Table 3:** Echocardiographic and invasive characteristics according to the echocardiographic and integrative staging.

	Total population (*n* = 90)	Echo stages 0–2 (*n* = 41)	Echo stage 3 (*n* = 10)	Echo stage 4 (*n* = 39)	*p*-value	Integrative stages 0–2 (*n* = 38)	Integrative stage 3 (*n* = 30)	Integrative stage 4 (*n* = 22)	*p*-value
Echocardiographic data									
SBP (mmHg)	127.3 ± 14.3	131.3 ± 13.6	130.6 ± 13.3	122.2 ± 14.0	**0**.**02**	129.9 ± 11.8	130.4 ± 15.0	119.0 ± 14.5	**0**.**01**
DBP (mmHg)	68.5 ± 8.2	70.0 ± 8.2	68.7 ± 7.4	66.8 ± 8.4	0.24	68.1 ± 8.8	70.7 ± 7.0	64.8 ± 7.9	0.10
HR (bpm)	72.8 ± 12.5	73.0 ± 14.4	76.9 ± 14.2	71.4 (9.8)	0.54	72.0 ± 13.0	75.2 ± 13.9	70.8 ± 9.1	0.47
LVMi (g/m^2^)	124.3 ± 36.6	124.7 ± 34.5	143.6 ± 18.9	118.7 ± 41.1	0.5	118.6 ± 33.3	130.8 ± 29.6	125.6 ± 50.2	0.12
SVi (ml/m^2^)	42.3 ± 9.6	45.1 ± 9.2	48.2 ± 9.6	37.9 ± 8.3	**<0**.**01**	44.6 ± 9.3	41.5 ± 10.1	39.0 ± 8.7	0.11
LVEF (%)	54.7 ± 10.7	56.9 ± 8.2	60.6 ± 7.8	50.8 ± 12.3	**0**.**02**	56.3 ± 9.2	56.0 ± 9.3	49.9 ± 13.5	0.24
E/E′ mean	16.2 ± 6.4	16.2 ± 6.7	19.9 ± 8.6	15.5 ± 5.4	0.42	16.0 ± 6.3	16.5 ± 7.4	16.3 (5.2)	0.85
LAVi (ml/m^2^)	52.6 ± 18.8	46.1 ± 14.9	65.8 ± 22.0	56.8 ± 19.6	**<0**.**01**	45.1 ± 14.4	58.1 ± 20.7	59.6 ± 19.0	**<0**.**01**
Peak aortic valve velocity (m/s)	4.2 ± 0.7	4.4 ± 0.6	4.3 ± 0.7	4.0 ± 0.6	**0**.**03**	4.2 ± 0.6	4.3 ± 0.8	4.2 ± 0.6	0.93
Aortic mean gradient (mmHg)	45.9 ± 15.0	49.7 ± 15.5	45.7 ± 15.4	41.9 ± 13.6	0.15	45.4 ± 14.1	46.9 ± 17.3	45.4 ± 13.7	0.92
AVA (cm^2^)	0.75 ± 0.16	0.75 ± 0.15	0.78 ± 0.15	0.74 ± 0.18	0.77	0.79 ± 0.13	0.71 ± 0.18	0.74 ± 0.19	0.17
AVAi (cm^2^/m^2^)	0.42 ± 0.09	0.43 ± 0.08	0.44 ± 0.10	0.40 ± 0.09	0.22	0.45 ± 0.07	0.40 ± 0.10	0.39 ± 0.08	**0**.**04**
MR moderate or severe	17 (18.9)	4 (9.8)	5 (50.0)	8 (20.5)	**0**.**02**	2 (5.3)	9 (30.0)	6 (27.3)	**0**.**01**
TR moderate or severe	18 (20.0)	0 (0.0)	9 (90.0)	9 (24.3)	**<0**.**01**	0 (0.0)	13 (43.3)	5 (25.0)	**<0**.**01**
RV basal diameter (mm)	39.1 ± 7.1	36.2 ± 5.1	43.4 ± 7.3	41.1 ± 7.7	**0**.**01**	36.6 ± 5.1	38.7 ± 6.7	44.8 ± 7.9	**<0**.**01**
TAPSE (mm)	17.9 ± 4.3	21.3 ± 3.0	17.7 ± 3.0	14.5 ± 2.7	**<0**.**01**	19.8 ± 3.7	18.9 ± 4.1	13.5 ± 1.4	**<0**.**01**
S′ (cm/s)	10.1 ± 2.2	11.2 ± 1.7	11.3 ± 2.4	8.6 ± 1.7	**<0**.**01**	10.8 ± 1.8	10.9 ± 2.1	7.7 ± 0.9	**<0**.**01**
RV FAC (%)	40 ± 12	47 ± 7	42 ± 4	32 ± 12	**<0**.**01**	46 ± 9	42 ± 9	28 ± 9	**<0**.**01**
sPAP (mmHg)	45.2 ± 15.3	36.6 ± 8.4	63.9 ± 16.9	49.2 ± 14.8	**<0**.**01**	35.8 ± 7.2	50.0 ± 17.2	54.8 ± 14.2	**<0**.**01**
Invasive data									
RAP (mmHg)	6.2 ± 4.5	4.5 ± 3.5	8.5 ± 4.9	7.3 ± 4.7	**0**.**01**	3.7 ± 2.3	7.5 ± 4.4	8.5 ± 5.2	**<0**.**01**
sPAP (mmHg)	44.8 ± 18.9	37.6 ± 15.3	59.0 ± 22.7	48.7 ± 18.6	**<0**.**01**	30.6 ± 6.4	53.2 ± 17.7	57.9 ± 19.0	**<0**.**01**
dPAP (mmHg)	15.6 ± 8.7	13.3 ± 7.7	19.8 ± 7.6	17.1 ± 9.3	**0**.**03**	9.2 ± 4.3	19.7 ± 7.0	21.3 ± 9.3	**<0**.**01**
mPAP (mmHg)	25.4 ± 11.6	21.4 ± 10.0	32.9 ± 12.0	27.7 ± 11.9	**0**.**01**	16.3 ± 4.4	30.9 ± 10.0	33.5 ± 12.0	**<0**.**01**
PCWP (mmHg)	16.1 ± 9.2	13.3 ± 7.6	23.0 ± 8.9	17.6 ± 9.7	**0**.**01**	10.1 ± 3.6	20.6 ± 8.2	21.0 ± 11.0	**<0**.**01**
Cardiac output (L/min)	4.8 ± 1.2	5.0 ± 1.1	5.0 ± 1.4	4.4 ± 1.1	**0**.**02**	5.0 ± 1.2	4.7 ± 1.1	4.4 ± 1.3	0.06
Cardiac index (L/min/m^2^)	2.7 ± 0.7	2.9 ± 0.6	2.8 ± 0.8	2.5 ± 0.6	**<0**.**01**	2.8 ± 0.6	2.8 ± 0.6	2.4 ± 0.7	**0**.**03**
SVi (ml/min/m^2^)	38.8 ± 9.4	41.5 ± 9.5	39.3 ± 6.2	35.8 ± 9.2	**0**.**02**	41.3 ± 9.0	38.7 ± 8.1	34.5 ± 10.5	**0**.**02**
PVR (UW)	2.0 ± 1.6	1.6 ± 1.1	1.9 ± 1.2	2.4 ± 2.0	0.11	1.3 ± 0.6	2.4 ± 1.7	2.8 ± 2.1	**<0**.**01**

Values are given as mean ± standard deviation or *n* (%).

Bold indicates statistically significant *p*-values (*p* < 0.05).

SBP, systolic blood pressure; DBP, diastolic blood pressure; HR, heart rate; LVMi, left ventricular mass index; SVi, stroke volume index; LVEF, left ventricular ejection fraction; LAVI, left atrial volume index; AVA, aortic valve area; AVAi, aortic valve area index; MR, mitral regurgitation; TR, tricuspid regurgitation; RV, right ventricular; TAPSE, tricuspid annular plane systolic excursion; S′, tissue Doppler derived systolic movement of the RV lateral wall; FAC, fractional area change; sPAP, systolic pulmonary pressure; RAP, right atrial pressure; dPAP, diastolic pulmonary artery pressure; mPAP, mean pulmonary pressure; PCWP, pulmonary capillary wedge pressure; PVR, Pulmonary vascular resistance.

In comparison with the patients in stages 0–2 or stage 3 in the echocardiographic staging system, the patients in stage 4 had significantly lower LV ejection fraction (56.9 ± 8.2% in stages 0–2, 60.6 ± 7.8% in stage 3, 50.8 ± 12.3% in stage 4, *p* = 0.02), stroke volume index (45.1 ± 9.2 ml/m^2^ in stages 0–2, 48.2 ± 9.6 ml/m^2^, 37.9 ± 8.3 ml/m^2^, *p* < 0.01), and peak aortic velocity (4.4 ± 0.6 in stages 0–2, 4.3 ± 0.7 in stage 3, 4.0 ± 0.6 m/s; *p* = 0.03). As expected, the stage 4 patients more often had RV dilatation and dysfunction according to both staging classifications. Stages 3 and 4 were also associated with larger left atrial volumes, significant mitral and tricuspid regurgitations, and higher sPAP.

### 3.3. Baseline invasive data

The baseline invasive data for the overall population and according to the stage of cardiac injury are summarized in Table 3. In the integrative staging, a progressive increase in measured invasive pressures (right atrial pressure, systolic, diastolic, and mean pulmonary arterial pressure, and pulmonary capillary wedge pressure) and a corresponding reduction in cardiac index (2.8 ± 0.6 L/min/m^2^ in stages 0–2, 2.8 ± 0.6 L/min/m^2^ in stage 3, 2.4 ± 0.7 L/min/m^2^ in stage 4; *p* = 0.03) and stroke volume index (41.3 ± 9.0 ml/m^2^ in stages 0–2, 38.7 ± 8.1 ml/m^2^ in stage 3, 34.5 ± 10.5 ml/m^2^ in stage 4; *p* = 0.02) were observed across all the spectrum of stages.

As expected, the use of RHC led to an increase in the identification of patients with PH in comparison with echocardiography, which translates into three times higher number of patients in stage 3 seen in the integrative staging as compared with the echocardiography staging system. Intriguingly, echocardiography was able to accurately identify the patients in low-flow state as well as the invasive method (cardiac index 2.9 ± 0.6 L/min/m^2^ in stages 0–2, 2.8 ± 0.8 L/min/m^2^ in stage 3, 2.5 ± 0.6 L/min/m^2^ in stage 4, *p* < 0.01, and stroke volume index 41.5 ± 9.5 ml/m^2^ in stages 0–2, 39.3 ± 6.2 ml/m^2^ in stage 3, 35.8 ± 9.2 ml/m^2^ in stage 4, *p* = 0.02). As expected, the integrative staging was the only one able to show differences between groups in terms of pulmonary vascular resistance (1.3 ± 0.6 WU in stages 0–2, 2.4 ± 1.7 WU in stage 3, 2.8 ± 2.1 WU in stage 4, *p* < 0.01).

### 3.4. Clinical outcomes

The clinical outcomes for the entire population and by stages are summarized in Table 4. During a median follow-up of 2.9 (1.5–3.9) years, 43 patients (47.8%) died, of which 19 (21.1%) from cardiovascular causes, and 29 patients (32.2%) had a cardiac-related hospitalization. According to the integrative staging, there was a significant difference in terms of all-cause mortality (34.2% in stages 0–2, 46.7% in stage 3, 72.7% in stage 4; *p* = 0.02) and cardiovascular death (7.9% in stages 0–2, 20.0% in stage 3, 45.5% in stage 4; *p* < 0.01) across all stages. The echocardiographic staging showed higher and comparable all-cause mortality and cardiovascular mortality in stages 3 and 4 (*p* = 0.57 and *p* = 0.13, respectively). Other MACE (stroke, cardiac-related hospitalization, and myocardial infarction) and NYHA functional class III/IV showed no difference between the two groups in both staging models.

**Table 4:** Clinical outcomes after TAVI.

	Total population (*n* = 90)	Echo stages 0–2 (*n* = 41)	Echo stage 3 (*n* = 10)	Echo stage 4 (*n* = 39)	*p*-value	Integrative stages 0–2 (*n* = 38)	Integrative stage 3 (*n* = 30)	Integrative stage 4 (*n* = 22)	*p*-value
All-cause mortality	43 (47.8)	17 (41.5)	5 (50.0)	21 (53.8)	0.57	13 (34.2)	14 (46.7)	16 (72.7)	**0**.**02**
Cardiovascular death	19 (21.1)	5 (12.2)	3 (30.0)	11 (28.2)	0.13	3 (7.9)	6 (20.0)	10 (45.5)	**<0**.**01**
Cardiac-related hospitalization	29 (32.2)	13 (31.7)	4 (40.0)	12 (30.8)	0.90	13 (34.2)	12 (40.0)	4 (18.2)	0.54
Stroke	10 (11.1)	7 (17.1)	2 (20.0)	1 (2.6)	0.07	6 (15.8)	3 (10.0)	1 (4.5)	0.58
Myocardial infarction	3 (3.3)	0 (0.0)	0 (0.0)	3 (7.7)	0.16	2 (5.3)	0 (0.0)	1 (4.5)	0.42
NYHA class III/IV	7 (7.8)	3 (7.3)	1 (10.0)	3 (7.7)	0.86	4 (10.5)	3 (10.0)	0 (0.0)	0.73

Values are given as *n* (%).

Bold indicates statistically significant *p*-values (*p* < 0.05).

NYHA, New York Heart Association.

### 3.5. Survival analysis

**Figure 3:**
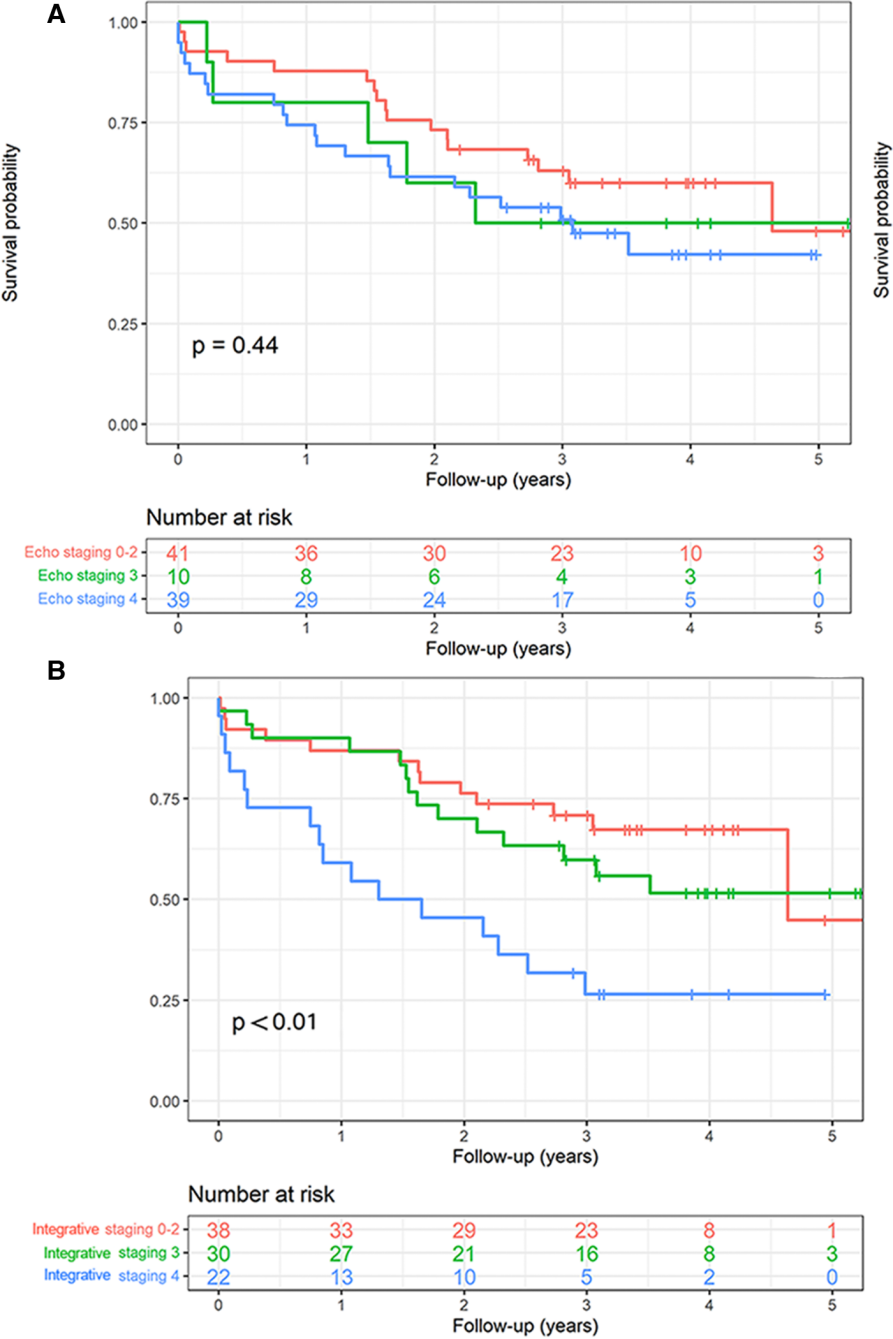
The Kaplan–Meier survival curve analysis of the integrative and echocardiographic staging. (**A**) The echocardiographic staging did not demonstrate any significant difference in terms of survival probability between the stages (log-rank *p* = 0.44); (**B**) the Kaplan–Meier curve analysis of the integrative staging evidenced a significant lower survival probability with advancing stages of cardiac damage (log-rank *p* < 0.01).

The Kaplan–Meier curve analysis of the integrative staging (Figure 3B) showed a significantly lower survival probability with advanced stages of cardiac damage (*p* < 0.01). Stage 4 showed a significantly lower survival probability compared with stages 0–2 (*p* < 0.01, coeff 3.15, 95% CI 1.51–6.58) and stage 3 (*p* = 0.03, coeff 2.32, 95% CI: 1.13–4.78). On the contrary, stage 3 did not show a significantly lower survival probability compared with stages 0–2 (*p* = 0.44, coeff: 1.36, 95% CI: 0.64–2.89). The intersection of the curves of stages 0–2 and 3 was likely related to the small number of patients present after the 4-year follow-up. The echocardiographic staging (Figure 3A) of our cohort did not reveal any significant difference in terms of survival probability between stages (*p* = 0.44).

### 3.6. Prognostic value of the two models

In a multivariable Cox survival model, the integrative staging emerged as a strong independent predictor of all-cause mortality for each stage increase [adjusted hazard ratio (HR): 1.69; 95% confidence interval 1.16–2.44; *p* < 0.01] after adjustment for several variables known for their clinical relevance (Table 5). In contrast, the echocardiographic staging was not associated with all-cause mortality in univariable cox survival analysis (HR: 1.13; 95% confidence interval 0.84–1.51; *p* = 0.43).

**Table 5:** Univariable and multivariable Cox proportional hazard analysis.

	Univariable analysis		Multivariable analysis	
	Hazard ratio (95% CI)	*p*-value	Adjusted hazard ratio (95% CI)	Adjusted *p*-value
NYHA class III/IV, yes/no	**1.97 (1.04–3.74)**	**0.04**	1.89 (0.97–3.68)	0.06
Chronic kidney disease, eGFR < 60 ml/min/1.73 m^2^, yes/no	1.06 (0.54–2.06)	0.87	1.37 (0.65–2.89)	0.41
Diabetes, yes/no	0.70 (0.35–1.40)	0.31	0.76 (0.36–1.58)	0.46
History of atrial fibrillation, yes/no	1.07 (0.59–1.94)	0.83	0.59 (0.29–1.23)	0.16
Age, per 1 year increase	0.95 (0.92–1.01)	0.16	0.95 (0.90–1.00)	0.05
Peak aortic valve velocity, per 1 m/s increase	0.80 (0.50–1.30)	0.38	0.64 (0.38–1.09)	0.10
Integrative cardiac damage staging, per 1 stage increase	**1.58 (1.11–2.23)**	**0.01**	**1.69 (1.16–2.44)**	**<0.01**
Echocardiographic cardiac damage staging, per 1 stage increase	1.13 (0.84–1.51)	0.43	—	—

Bold indicates the statistically significant *p*-values and adjusted *p*-values (*p* < 0.05) and the corresponding hazard ratio (95% CI) and adjusted hazard ratio (95% CI).

CI, confidence interval; NYHA, New York Heart Association; eGFR, estimated glomerular filtration rate.

## 4. Discussion

To the best of our knowledge, this is the first study to evaluate an AS staging integrating both echocardiographic and invasive parameters in a cohort of patients with severe symptomatic AS undergoing TAVI. In the present study, in the integrative cardiac damage staging, there was a gradual increase in all-cause and cardiovascular mortality per each increase of cardiac damage stage, whereas in the echocardiographic staging, stages 3 and 4 had similar mortality rates. Analyzing the Kaplan–Meier curves, the integrative stage 4 showed a significantly lower survival probability compared with stages 0–2 and 3. Also, the new proposed integrative staging has shown to be a predictor for all-cause death in multivariable analysis, above and beyond several clinical factors known to have a negative prognostic impact. On the other hand, in our study, the echocardiographic staging was not correlated with all-cause or cardiovascular mortality. Therefore, we can hypothesize that the integrative staging might allow for better individual risk stratification as compared with the conventional echocardiographic staging model. These results reinforce the interest of using the RHC in the workup of patients with advanced AS.

### 4.1. Integrative cardiac damage staging

Recently, an echocardiographic staging classification, including 4–5 stages, has been proposed and validated to assess and report the extent of cardiac damage associated with AS. A gradual increase in mortality was observed for each stage increment in symptomatic patients with severe AS undergoing AVR, as well as in asymptomatic patients with moderate or severe AS. Advanced stages were associated with a marked increase in the short-term risk of mortality both before and after AVR (4–8). In their invasive study, Maeder et al. (10) have shown that a staging system of cardiac damage based solely on invasive hemodynamic parameters could be used to predict mortality, in patients with severe AS undergoing AVR. In this study, the patients with stages 1 (increased LV end-diastolic pressure) and 2 (increased pulmonary capillary wedge pressure) had a similar prognosis to the patients without cardiac lesion (stage 0), whereas those with increased right cardiac pressures (PH and right atrial pressure) were at greater risk of cardiovascular events. Interestingly, Maeder et al. did not recommend carrying out an invasive left hemodynamic evaluation, which is not without risk and may be harmful (10, 15).

Our study strongly supports the use of RHC for the evaluation of cardiac damage and provides additional data on the importance of further examining the upstream repercussions of AS on the pulmonary circulation and the right ventricle. We found that a high proportion of patients undergoing TAVI had PH (46%) or RV dysfunction (39%), and these patients were at high risk for adverse outcomes. With the integrative approach, the gradual increase in mortality for each stage was more pronounced compared with the conventional echocardiographic approach. The risk of death was even more marked in stage 4 when the variables were analyzed as a function of time. The two staging classifications differed mainly by a greater number of patients with definite PH and a better characterization of patients with a low-flow state using RHC data.

Although a possible explanation for the lack of correlation between the echocardiographic staging system and outcome in our study as opposed to the previously mentioned studies could be related to the low number of patients included in our study, the use of RHC could prove to be useful for the evaluation of these patients. RHC represents the gold standard for measuring pulmonary pressures, defining and classifying PH (16), and for accurately evaluating the flow state of the patient in terms of cardiac output. In the clinical context of evaluating patients before AVR, the correct measurement of pulmonary pressures is crucial because PH (defined as mPAP ≥ 25 mmHg) pre- and post-TAVI has been shown to have a strong impact on long-term survival after AVR (17, 18). In our study, in the integrative staging system, we defined PH as a mPAP ≥ 25 mmHg, as this was the cut-off value recommended by the previous guidelines on PH and because this value has been shown to be associated with long-term survival after AVR in patients with AS (11, 17, 18). The latest guidelines on PH, which have been published after the completion of our study, recommend decreasing the cut-off value of mPAP to >20 mmHg for defining PH, based on the data in normal patients and certain specific patient populations such as patients with pulmonary fibrosis or systemic sclerosis (16). To the best of our knowledge, there are no studies showing a decrease in prognosis in AS patients with mPAP ≥ 20 mmHg, and although the use of a lower cut-off would lead to an increase in the number of patients diagnosed with PH, whether or not this would translate to an improved outcome prediction in AS patients remains to be determined.

The definition of PH in the echocardiographic staging systems is only based on an sPAP ≥ 60 mmHg (4–8). As such, the echocardiographic staging systems only identify patients with a high probability of PH, missing out a large number of patients who are actually at increased risk of worse outcome. In our study, RHC led to three times increase in the number of patients in stage 3 as compared with the echocardiographic staging. Although echocardiography is often the first exam used for evaluating the presence of PH, it can only estimate the probability of PH as low, intermediate, or high, based on the value of the maximum velocity of the tricuspid regurgitant jet and the presence of additional signs suggestive of PH (16). The latest guidelines on PH stress the importance of not relying on a single echocardiographic parameter for estimating the probability of PH. In the presence of indirect signs of PH, the probability of PH moves to the next category (from low to intermediate and from intermediate to high probability) (16). The use of a multiparametric approach for estimating the probability of PH would without a doubt lead to an increase in the number of patients with AS and PH, as compared with the current staging systems. However, RHC is the sole exam based on which a definite diagnosis of PH can be made. It would seem thus reasonable to consider the performance of RHC in patients with intermediate or high probability of PH based on a multiparametric echocardiographic evaluation. Whether or not the use of RHC, which is not without risks, in patients with an intermediate or high echocardiographic probability of PH could lead to an improvement in outcome prediction as compared with the echocardiographic evaluation alone remains to be evaluated by future studies.

### 4.2. Clinical implications

With the aging of the population and the advent of TAVI as a treatment option, clinicians are increasingly confronted with patients presenting with long-standing AS, multimorbidity, progressive RV dysfunction, and pulmonary vascular disease. Although operative mortality is increased in cases of severe PH, the benefit provided by surgical AVR is undoubtedly greater than conservative management (19). However, several recent studies have reported that worsening of RV function was more common after surgical AVR than after TAVI, which seemed to be associated with an increased risk of mortality (20). RV function and RV–PA coupling even tended to improve after TAVI. The prognosis of patients is therefore significantly impacted in the presence of PH or RV dysfunction, which can condition the type of AVR procedure. Therefore, TAVI may be preferred in patients with cardiac damage stage ≥3, that is, with pre-existing PH, ≥ moderate tricuspid regurgitation and/or RV dysfunction. In our study, despite a high surgical risk, a significant proportion of patients (approximately 50%) were at stage ≤2 according to the integrative classification; these patients had a better prognosis than those in stages 3–4. Therefore, the presence of an advanced cardiac stage underlines both the need for careful follow-up after TAVI and the integration of staging into the decision-making process before intervention.

### 4.3. Limitations

First, the number of patients examined was relatively low, especially with initial stages 0–1, which is why they were logically grouped with stage 2, corresponding to left-chamber cardiac involvement. However, there are relatively few published data on invasive hemodynamics collected systematically from real-life patients with AS before TAVI. Second, echocardiography and RHC were not performed simultaneously. Nonetheless, they were both done within the month of the TAVI procedure, and we do not expect the cardiac stage to change in such a short time. Third, the staging classification was assessed in patients with severe AS treated by TAVI. Therefore, we did not assess the impact of the extent of cardiac damage during the natural course of AS.

### 4.4. Conclusion

Staging of extra-valvular cardiac damage using both echocardiographic and invasive parameters was independently associated with a progressive increase in adverse outcomes following TAVI for severe AS. This integrative staging might allow for better individual risk stratification compared with the conventional echocardiographic staging model. Future prospective studies are needed to assess the additional value of this integrative cardiac damage staging system during the natural course of AS.

## Data Availability

The raw data supporting the conclusions of this article will be made available by the authors, without undue reservation.
