# Supplementary material for: A new integrative approach combining right heart catheterization and echocardiography to stage aortic stenosis-related cardiac damage
Source: Front Cardiovasc Med. 2023 Aug 2;10:1184308. doi: 10.3389/fcvm.2023.1184308 (PMC10436206; doi:10.3389/fcvm.2023.1184308)
Supplement: Supplementary file 1 [file Datasheet1.docx]

**Supplementary Table 1: Baseline demographic and clinical characteristics**

Values are given as *n* (%) or as mean ± standard deviation.

PCI: percutaneous coronary intervention, CABG: coronary artery bypass grafting, COPD: chronic obstructive pulmonary disease, CKD: Chronic kidney disease, eGFR: estimated glomerular filtration rate, NYHA: New York Heart association, CCS: Canadian Cardiovascular Society, STS: Society of Thoracic Surgeons, ACE: Angiotensin-converting enzyme, ARBs: Angiotensin receptors blockers, MRA: Mineralcorticoid receptor antagonist, SAPT: single antiplatelet therapy, DAPT: dual antiplatelet therapy.

|  | Total population (n=90) | Echo stages 0-2 (n=41) | Echo stage 3 (n=10) | Echo stage 4 (n=39) | p-value | Integrative  stages 0-2  (n=38) | Integrative stage 3  (n=30) | Integrative stage 4 (n=22) | p-value |
| --- | --- | --- | --- | --- | --- | --- | --- | --- | --- |
| Age (Years) | 82.2 ± 5.5 | 82.3 ± 6.4 | 85.7 ± 2.5 | 81.3 ± 4.9 | **0.03** | 82.2 ± 6.3 | 82.5 ± 5.5 | 81.8 ± 4.4 | 0.74 |
| Female | 50 (55.6) | 23 (56.1) | 5 (50.0) | 22 (56.4) | 0.91 | 21 (55.3) | 17 (56.7) | 12 (54.5) | 0.99 |
| BMI | 26.5 ± 4.9 | 25.6 ±4.6 | 26.4 ± 6.0 | 27.5 ± 5.0 | 0.10 | 26.2 ± 5.1 | 26.4 ± 5.0 | 27.0 ± 4.8 | 0.41 |
| **Cardiad risk factors** | | | | | | | | | |
| Current smoker | 21 (23.3) | 13 (31.7) | 0 (0.0) | 8 (20.5) | 0.08 | 12 (31.6) | 6 (20.0) | 3 (13.6) | 0.25 |
| Hypertension | 82 (91.1) | 36 (87.8) | 10 (100.0) | 36 (92.3) | 0.66 | 34 (89.5) | 29 (96.7) | 19 (86.4) | 0.36 |
| Dyslipedemia | 67 (74.4) | 31 (75.6) | 7 (70.0) | 29 (74.4) | 0.94 | 29 (76.3) | 23 (76.7) | 15 (68.2) | 0.74 |
| Diabetes | 29 (32.2) | 10 (24.4) | 2 (20.0) | 17 (43.6) | 0.14 | 14 (36.8) | 6 (20.0) | 9 (40.9) | 0.20 |
| **Past medical history** | | | | | | | | | |
| Coronary Artery Disease | 59 (65.6) | 27 (65.9) | 6 (60.0) | 26 (66.7) | 0.90 | 26 (68.4) | 19 (63.3) | 14 (63.6) | 0.89 |
| Recent Myocardial Infarction | 3 (3.3) | 2 (4.9) | 0 (0.0) | 1 (2.6) | >0.99 | 0 (0.0) | 2 (6.7) | 1 (4.5) | 0.34 |
| Previous PCI | 37 (41.6) | 18 (43.9) | 3 (33.3) | 16 (41.0) | 0.86 | 15 (39.5) | 14 (48.3) | 8 (36.4) | 0.65 |
| Previous CABG | 21 (23.3) | 8 (19.5) | 2 (20.0) | 11 (28.2) | 0.68 | 8 (21.1) | 6 (20.0) | 7 (31.8) | 0.55 |
| Previous cardiac surgery other than CABG | 2 (2.2) | 0 (0.0) | 0 (0.0) | 2 (5.1) | 0.40 | 8 (21.1) | 6 (20.0) | 7 (31.8) | 0.55 |
| Previous Stroke | 13 (14.4) | 5 (12.2) | 2 (20.0) | 6 (15.4) | 0.69 | 4 (10.5) | 4 (13.3) | 5 (22.7) | 0.44 |
| Previous vascular disease | 41 (45.6) | 19 (46.3) | 1 (10.0) | 21 (53.8) | **0.04** | 20 (52.6) | 11 (36.7) | 10 (45.5) | 0.42 |
| COPD | 19 (21.1) | 8 (19.5) | 1 (10.0) | 10 (25.6) | 0.58 | 8 (21.1) | 5 (16.7) | 6 (27.3) | 0.68 |
| CKD, eGFR <60 mL/min/1,73m2) | 64 (71.1) | 26 (63.4) | 7 (70.0) | 31 (79.5) | 0.27 | 24 (63.2) | 22 (73.3) | 18 (81.8) | 0.29 |
| **Baseline cardiac rhythm** | | | | | | | | | |
| Atrial Fibrillation | 40 (44.4) | 11 (26.8) | 6 (60.0) | 23 (59.0) | **0.01** | 10 (26.3) | 18 (60.0) | 12 (54.5) | **0.01** |
| Permanent Pacemaker | 15 (16.7) | 3 (7.3) | 3 (30.0) | 9 (23.1) | 0.05 | 6 (15.8) | 5 (16.7) | 4 (18.2) | >0.99 |
| **Symptoms** | | | | | | | | | |
| NYHA class III or IV | 49 (55.1) | 16 (40.0) | 4 (40.0) | 29 (74.4) | **0.01** | 16 (43.2) | 18 (60.0) | 15 (68.2) | 0.14 |
| CCS class II or IV | 4 (4.4) | 2 (4.9) | 0 (0.0) | 2 (5.1) | >0.99 | 2 (5.3) | 2 (6.7) | 0 (0.0) | 0.68 |
| **Risk assessment** | | | | | | | | | |
| Euroscore II (%) | 6.2 ± 5.1 | 5.6 ± 5.2 | 5.0 ± 2.2 | 7.0 ± 5.4 | 0.35 | 5.7 ± 5.3 | 6.1 ± 3.9 | 7.1 ± 6.2 | 0.30 |
| STS (%) | 4.9 ± 2.7 | 4.9 ± 3.2 | 5.9 ± 2.8 | 4.6 ± 1.9 | 0.34 | 4.43 ± 2.6 | 5.64 ± 3.1 | 4.55 ± 2.0 | 0.25 |
| **Medical therapy** | | | | | | | | | |
| Diuretic | 60 (66.7) | 24 (58.5) | 8 (80.0) | 28 (71.8) | 0.31 | 23 (60.5) | 21 (70.0) | 16 (72.7) | 0.56 |
| Beta blockers | 60 (66.7) | 26 (63.4) | 7 (70.0) | 27 (69.2) | 0.86 | 22 (57.9) | 24 (80.0) | 14 (63.6) | 0.15 |
| ACE inhibitors | 27 (30.0) | 11 (26.8) | 1 (10.0) | 15 (38.5) | 0.19 | 8 (21.1) | 9 (30.0) | 10 (45.5) | 0.14 |
| ARBs | 25 (27.8) | 12 (29.3) | 4 (40.0) | 9 (23.1) | 0.56 | 12 (31.6) | 9 (30.0) | 4 (18.2) | 0.51 |
| MRA | 11 (12.2) | 5 (11.6) | 2 (20.0) | 4 (10.8) | 0.73 | 4 (8.7) | 6 (20.0) | 1 (7.1) | 0.50 |
| SAPT | 43 (47.8) | 19 (46.3) | 2 (20.0) | 22 (56.4) | 0.11 | 12 (31.6) | 9 (30.0) | 4 (18.2) | 0.51 |
| DAPT | 16 (17.8) | 8 (19.5) | 1 (10.0) | 7 (17.9) | 0.93 | 7 (18.4) | 6 (20.0) | 3 (13.6) | 0.88 |

**Supplementary Table 2: Intra-procedural and post-procedural characteristics.**

Values are given as n (%) or median (25th percentile - 75th percentile).

TAVR: transcatheter aortic valve replacement, PPM: pacemaker implantation

|  | Total population (n=90) | Echo stages 0-2 (n=41) | Echo stage 3 (n=10) | Echo stage 4 (n=39) | p-value | Integrative stages 0-2  (n=38) | Integrative stage 3  (n=30) | Integrative stage 4 (n=22) | p-value |
| --- | --- | --- | --- | --- | --- | --- | --- | --- | --- |
| **Type of valve**  0.716 0.716 | | | | | | | | | |
| Corevalve (Medtronic) | 75 (83.3) | 35 (85.4) | 9 (90.0) | 31 (79.5) |  | 32 (84.2) | 25 (83.3) | 18 (81.8) |  |
| Evolut R (Medtronic) | 15 (16.7) | 6 (14.6) | 1 (10.0) | 8 (20.5) |  | 6 (15.8) | 5 (16.7) | 4 (18.2) |  |
| **Approach**  0.112 0.672 | | | | | | | | | |
| Femoral | 67 (74.4) | 28 (68.3) | 10 (100.0) | 29 (74.4) |  | 25 (65.8) | 24 (80.0) | 18 (81.8) |  |
| Subclavian | 18 (20.0) | 12 (29.3) | 0 (0) | 6 (15.4) |  | 10 (26.3) | 5 (16.7) | 3 (13.6) |  |
| Other | 5 (5.6) | 1 (2.4) | 0 (0) | 4 (10.3) |  | 3 (7.9) | 1 (3.3) | 1 (4.5) |  |
| **General anesthesia** | 60 (66.7) | 27 (65.9) | 7 (70.0) | 26 (66.7) | <0.999 | 24 (63.2) | 21 (70.0) | 15 (68.2) | 0.826 |
| **Periprocedural bleeding** | 15 (16.7) | 7 (17.1) | 1 (10.0) | 7 (17.9) | <0.999 | 8 (21.1) | 4 (13.3) | 3 (13.6) | 0.719 |
| **Post- TAVR need of PPM within 30 days** | 16 (18.2) | 7 (17.1) | 3 (30.0) | 6 (16.2) | 0.573 | 4 (10.5) | 8 (27.6) | 4 (19.0) | 0.195 |
| **Lenght of hospital stay (days)** | 8 (6-11) | 8 (6-11) | 9 (6-14) | 7 (5-10) | 0.594 | 7 (6-9) | 10 (7-12) | 8 (5-11) | 0.284 |

**Supplementary Table 3: Univariable Cox proportional Hazard analysis of the parameters included in the echocardiographic and integrative staging systems.**

CI: confidence interval, LVMi: left ventricular mass index, LVEF: left ventricular ejection fraction, LAVI: left atrial volume index, MR: mitral regurgitation, sPAP: systolic pulmonary pressure, TR: tricuspid regurgitation, TAPSE: tricuspid annular plane systolic excursion, s’: tissue Doppler derived systolic movement of the RV lateral wall, FAC: fractional area change, SVi: stroke volume index, mPAP: mean pulmonary pressure, RAP: right atrial pressure.

|  | **Univariable analysis** | |
| --- | --- | --- |
|  | Hazard ratio (95% CI) | p-value |
| **Echocardiographic data** | | |
| LVMi (g/m^2^) | 1.00 (0.99-1.01) | 0.973 |
| LV diastolic dysfunction ≥ 2 | 1.24 (0.67-2.29) | 0.492 |
| LVEF (%) | 0,98 (0,95-1,00) | 0.098 |
| LAVi (mL/m^2^) | 1.01 (0.99-1.02) | 0.570 |
| MR moderate or severe | 1.77 (0.90-3.45) | 0.097 |
| sPAP (mmHg) | 1.02 (1.00-1.03) | 0.111 |
| TR moderate or severe | 1.28 (0.63-2.60) | 0.498 |
| TAPSE (mm) | 0.93 (0.86-1.00) | **0.045** |
| S' (cm/s) | 0.89 (0.77-1.03) | 0.119 |
| RV FAC (%) | 0.08 (0.01-1.20) | 0.067 |
| SVi (mL/m^2^) | 0.99 (0.96-1.02) | 0.560 |
| **Invasive data** | | |
| mPAP (mmHg) | 1.03 (1.00-1.06) | **0.023** |
| Cardiac index (L/min/m^2^) | 0.70 (0.44-1.13) | 0.146 |
| RAP (mmHg) | 1.06 (0.99-1.13) | 0.102 |

**Supplementary Table 4: Univariable and Multivariable Cox proportional Hazard analysis of the integrative system including four clinical predictors.**

CI: confidence interval, NYHA: New York Heart association.

|  | **Univariable analysis** | | **Multivariable analysis (AIC = 340)** | |
| --- | --- | --- | --- | --- |
|  | Hazard ratio (95% CI) | p-value | Adjusted hazard ratio (95% CI) | Adjusted p-value |
| NYHA class III or IV, yes/no | **1.97 (1.04-3.74)** | **0.04** | 1.98 (1.02 -2.22) | **0.04** |
| Age, per 1 year increase | 0.95 (0.92-1.01) | 0.16 | 0.96 (0.91-1.01) | 0.09 |
| Peak aortic valve velocity, per 1 m/s increase | 0.80 (0.50-1.30) | 0.38 | 0.77 (0.48-1.23) | 0.27 |
| Integrative cardiac damage staging, per 1 stage increase | **1.58 (1.11-2.23)** | **0.01** | **1.57 (1.11-2.22)** | **0.01** |

**Supplementary Table 5: Univariable and Multivariable Cox proportional Hazard analysis of the echocardiographic system including four clinical predictors.**

CI: confidence interval, NYHA: New York Heart association.

|  | **Univariable analysis** | | **Multivariable analysis (AIC = 347)** | |
| --- | --- | --- | --- | --- |
|  | Hazard ratio (95% CI) | p-value | Adjusted hazard ratio (95% CI) | Adjusted p-value |
| NYHA class III or IV, yes/no | **1.97 (1.04-3.74)** | **0.04** | 2.09 (1.05-4.15) | **0.04** |
| Age, per 1 year increase | 0.95 (0.92-1.01) | 0.16 | 0.97 (0.92-1.01) | 0.17 |
| Peak aortic valve velocity, per 1 m/s increase | 0.80 (0.50-1.30) | 0.38 | 0.82 (0.50-1.35) | 0.44 |
| Echocardiographic cardiac damage staging, per 1 stage increase | 1.13 (0.84-1.51) | 0.43 | 0.99 (0.72-1.36) | 0.93 |
